# A clinical indicator-based prognostic model predicting treatment outcomes of pulmonary tuberculosis: a prospective cohort study

**DOI:** 10.1186/s12879-023-08053-x

**Published:** 2023-02-20

**Authors:** Mengyao Zhan, Hao Xue, Yuting Wang, Zhuchao Wu, Qin Wen, Xinling Shi, Jianming Wang

**Affiliations:** 1 Department of Epidemiology, Center for Global Health, School of Public Health, Nanjing Medical University, 101 Longmian Ave. Nanjing, 211166 Nanjing, China; 2 Department of Chronic Communicable Diseases, Yancheng Center for Disease Control and Prevention, 224002 Yancheng, China; 3 Department of Epidemiology, Gusu School, Nanjing Medical University, 211166 Nanjing, China

**Keywords:** Tuberculosis, Outcome, Biochemistry examination, Clinical indicator, Prognostic model

## Abstract

**Objectives:**

Identifying prognostic factors helps optimize the treatment regimen and promote favorable outcomes. We conducted a prospective cohort study on patients with pulmonary tuberculosis to construct a clinical indicator-based model and estimate its performance.

**Methods:**

We performed a two-stage study by recruiting 346 pulmonary tuberculosis patients diagnosed between 2016 and 2018 in Dafeng city as the training cohort and 132 patients diagnosed between 2018 and 2019 in Nanjing city as the external validation population. We generated a risk score based on blood and biochemistry examination indicators by the least absolute shrinkage and selection operator (LASSO) Cox regression. Univariate and multivariate Cox regression models were used to assess the risk score, and the strength of association was expressed as the hazard ratio (HR) and 95% confidence interval (CI). We plotted the receiver operating characteristic (ROC) curve and calculated the area under the curve (AUC). Internal validation was conducted by 10-fold cross-validation.

**Results:**

Ten significant indicators (PLT, PCV, LYMPH, MONO%, NEUT, NEUT%, TBTL, ALT, UA, and Cys-C) were selected to generate the risk score. Clinical indicator-based score (HR: 10.018, 95% CI: 4.904–20.468, *P* < 0.001), symptom-based score (HR: 1.356, 95% CI: 1.079–1.704, *P* = 0.009), pulmonary cavity (HR: 0.242, 95% CI: 0.087–0.674, *P* = 0.007), treatment history (HR: 2.810, 95% CI: 1.137–6.948, *P* = 0.025), and tobacco smoking (HR: 2.499, 95% CI: 1.097–5.691, *P* = 0.029) were significantly related to the treatment outcomes. The AUC was 0.766 (95% CI: 0.649–0.863) in the training cohort and 0.796 (95% CI: 0.630–0.928) in the validation dataset.

**Conclusion:**

In addition to the traditional predictive factors, the clinical indicator-based risk score determined in this study has a good prediction effect on the prognosis of tuberculosis.

**Supplementary Information:**

The online version contains supplementary material available at 10.1186/s12879-023-08053-x.

## Introduction

Tuberculosis (TB) continues to be a global concern and poses enormous threats to human health. In 2020, it caused 5.8 million new cases and 1.3 million deaths worldwide [1]. The current recommended antituberculosis treatment (ATT) regimen for drug-sensitive TB is a six-month regimen of four first-line drugs[2, 3], with a success rate of over 86%. Relapse rates varied across regions, ranging from about 3–10% in human immunodeficiency virus (HIV)-negative patients [4–7].

HIV infection, diabetes mellitus (DM), alcohol abuse, tobacco smoking, mental health have been recognized as risk factors for poor treatment outcomes[1, 8–11]. Substantial evidence has shown that age, body mass index (BMI), family income, and disease classification significantly affected the prognosis [9, 12–14]. Some studies have applied medical history and clinical symptom-based scores to estimate the prognosis of patients with TB, such as the Karnofsky Performance Status Scale [15] and the Charlson Comorbidity Index (CCI) [16]. Other studies have established demographic characteristics and laboratory test results, like race, acid-fast bacilli smear examination, albumin, white blood cell counts, hemoglobin, and C-reaction protein (CRP), to construct the predictive models [16–18].

Both baseline characteristics and dynamic clinical changes are related to the treatment outcomes. Identifying prognostic factors can help adjust for therapeutic regimens and intervention measures. In this study, we carried out a prospective cohort study on a group of patients with pulmonary TB to construct a clinical indicator-based risk score and estimate its ability to predict outcomes.

## Methods

### Study population

We performed a two-stage study by recruiting 346 pulmonary tuberculosis patients diagnosed between 2016 and 2018 in Dafeng city as the training cohort and 132 patients diagnosed between 2018 and 2019 in Nanjing city as the external validation population. Dafeng is located in the coastal area, and Nanjing is located in the southeast region of Jiangsu Province, China, respectively. The inclusion criteria were pulmonary TB patients who were clinically diagnosed according to the Diagnosis Criteria for Pulmonary Tuberculosis of China (WS 288–2017) and completed the baseline questionnaire investigation. Patients were excluded if they were (a) treated for < 1 month; (b) lost to follow-up; (c) HIV positive; or (d) diagnosed with other pulmonary diseases. The primary study end-point was the treatment failure or the relapse of TB, regarded as unfavorable treatment outcomes. Treatment failure was defined as bacteriologic or clinical failure, treatment interruption due to adverse drug reactions, transferring to multidrug-resistant therapy, or TB-related death. TB relapse was observed by following up until two years after the completion of ATT. This study defined favorable treatment outcomes for patients who were curative or completed the treatment without relapse within two years. The ethics committee of Nanjing Medical University approved this study. Written informed consent was obtained from study participants.

### Data collection and variable coding

We collected demographic and epidemiological information of study subjects using a structured questionnaire. Patients who were not treated previously or received ATT less than one month were defined as new cases; otherwise, they were retreatment patients with an ATT history. Clinical information was obtained through the hospital information system and laboratory tests, including therapeutic regimen, sputum smear tests, sputum culture results, the onset of symptoms, date of diagnosis, chest X-ray examination, blood tests, and treatment outcomes. If TB patients took ATT drugs regularly without interruption, it was defined as good treatment compliance; otherwise, it was described as poor treatment compliance. The delayed time of ATT included the patient’s and the doctor’s delays. The patient’s delay was calculated by the interval between the symptom occurrence and the date of seeking health care. The doctor’s delay was calculated by the gap between seeking health care and initiating treatment. We figured the chest X-ray (CXR) score based on the proportion of lung lesions and the occurrence of cavitation judged by at least two experienced clinicians. According to the literature reference [19], the CXR score= proportion of total lung affected×100 + 40 (if the cavity is present). Typical symptoms of TB included cough, expectoration, fever, weight loss, dyspnea, night sweats, hemoptysis, fatigue, and chest pain, and the score represented the number of symptoms. Blood and biochemical indicators included red blood cell (RBC), white blood cell (WBC), platelet (PLT), hemoglobin (HB), packed cell volume (PCV), red blood cell distribution width (RDW), absolute value of lymphocytes (LYMPH), percentage of lymphocytes (LYMPH%), monocyte absolute value (MONO), monocyte percentage (MONO%), absolute value of neutrophil (NEUT), neutrophilic percentage (NEUT%), absolute value of eosinophils (EOS), eosinophil percentage (EOS%), absolute value of basophils (BASO), basophil percentage (BASO%), total bilirubin (TBTL), alanine aminotransferase (ALT), aspartate aminotransferase (AST), urea (UREA), creatinine (Cr), uric acid (UA), cystatin C (Cys-C), and β2-microglobulin (β2-m). They were collected at the baseline routine examination before initiating ATT.

### Statistical analysis

Continuous variables were described as mean ± standard deviation (SD) or median together with interquartile range (IQR). Categorical variables were expressed as frequency and percentage. Continuous variables were compared by t-test if they were normally distributed; otherwise, they were compared by the Mann-Whitney test. Categorical variables were compared by chi-square test. Missing data of clinical data were imputed by multiple imputations shown in Supplementary materials [20, 21]. We applied the least absolute shrinkage and selection operator (LASSO) Cox regression analysis to screen clinical indices, extracted significant ones and their coefficients, and established a risk score system by predict function. The levels of clinical indicators were defined as an independent variable. The survival time and treatment outcomes of TB patients were considered as the response variables. The coefficients of each remained signature were derived from the LASSO regression analysis, and the risk score was generated using the formula of $risk score={\sum }_{i=1}^{n}\left({Coef}_{i}\text{*}{x}_{i}\right)$. Then we utilized the univariate Cox regression model to assess the risk score and built a predictive model. Furthermore, using the bidirectional stepwise method, we conducted a multivariable Cox regression model by considering the risk score, previously reported variables, or clinically relative variables [22].

The strength of association was expressed as the hazard ratio (HR) and 95% confidence interval (CI). We plotted a nomogram based on variables in the multivariate model and showed different weighted scores for each factor. The total points were the sum of scores, which could be transferred to the predictive probability of the individual outcome event according to function transformation between total points and prognostic situation. To evaluate the prediction effects of established models, we plotted receiver operating characteristic (ROC) curves and calculated the corresponding area under the curve (AUC) both in the training and validation cohorts. Harrell’s concordance index (C-index) was also used to assess the discrimination of models. Internal validation was constructed by the 10-fold cross-validation method. The Hosmer-Lemeshow test was applied to evaluate the model’s goodness of fit.

Data analyses were performed in R version 4.1.2, using the “mice”, “randomForest”, “rms”, “survival”, “survminer”, “My.stepwise”, “VIM”, “Hmisc”, “grid”, “lattice”, “Formula”, “pROC”, “caret” and “ResourceSelection” packages. *P* < 0.05 was regarded as statistically significant.

### Patient and public involvement

We collect anonymous information about TB patients. No individual patients or the public were involved in this study.

## Results

### Characteristics of study subjects

**Fig. 1:**
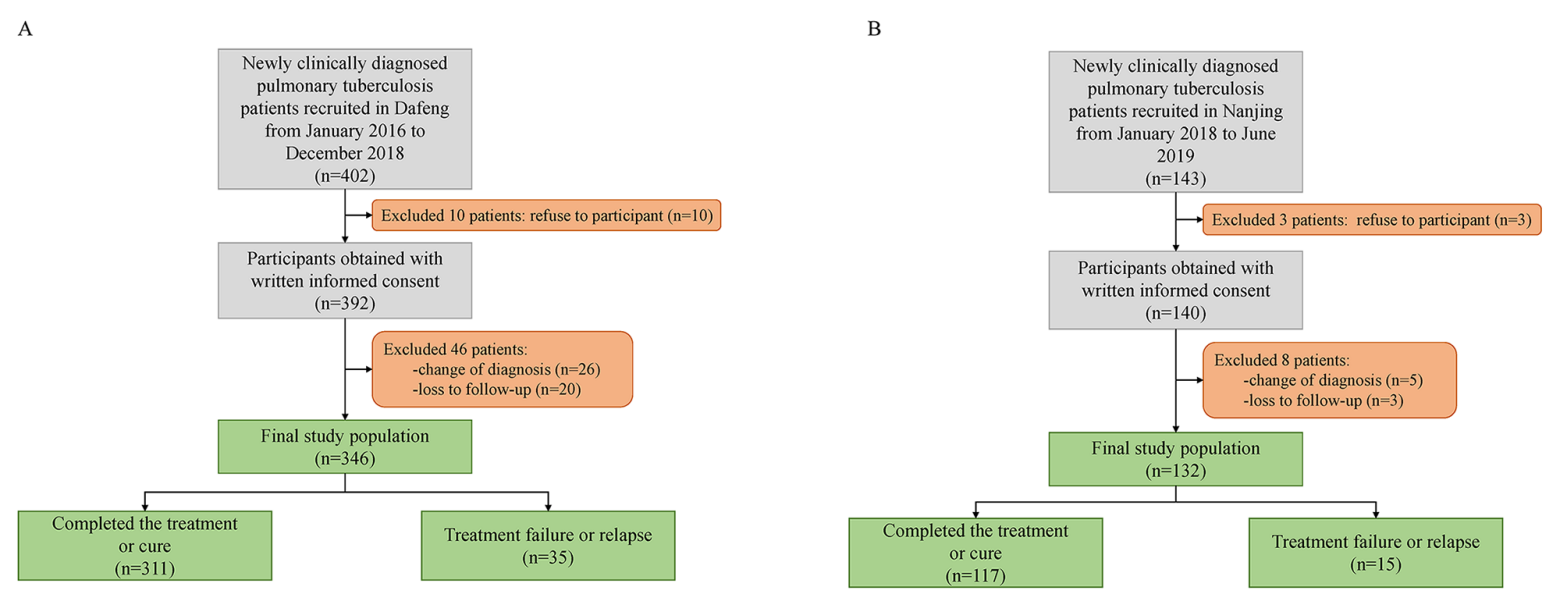
**Flowcharts of study participants with pulmonary tuberculosis enrolled in this study** Diagrams detailed inclusion and exclusion criteria and the numbers of participants excluded in training cohort (A) and validation cohort (B)

There were 402 pulmonary TB patients consecutively recruited into this study. Finally, 346 patients were kept in the training cohort dataset after excluding people who refused to participate (n = 10), diagnostic changes (n = 26), and loss to follow-up (n = 20) (Fig. 1A). There were 250 (72.3%) males and 96 (27.7%) females. Among them, 124 (35.8%) were over 60 years, 57 (16.5%) were underweight, 102 (29.5%) were ever-smokers, and 89 (25.7%) had an alcohol-drinking history. Nineteen (5.5%) subjects had an ATT history, and 34 (9.8%) were diagnosed with diabetes mellitus. The majority were farmers, and approximately half were low-income groups. Most received an ATT regimen of 2HRZE/4HR, while only 12 (3.5%) patients were treated with other regimens. Thirteen patients have an interruption of ATT (Table 1). Less than half were smear-positive. The median CXR score was 33.33, with an IQR of 40, of which 138 (39.9%) patients had pulmonary cavities. The mean symptom score was 3.39. The median time of the delayed treatment was 47 days, with an IQR of 71 days (Table 2). And the average time of follow-up was 190 days, ranging from 34 to 821 days. As a result, 35 subjects were observed with unfavorable outcomes during the follow-up, consisting of 11 adverse drug reactions, 4 TB-related deaths, 5 transferring to multidrug-resistant therapy, and 15 relapses after treatment.

**Table 1:** Characteristics of the study population

Characteristics	Training cohort N (%)	Validation group N (%)	*P* ^*^
Sex			0.006
Male	250 (72.3)	78 (59.1)	
Female	96 (27.7)	54 (40.9)	
Age			< 0.001
≤60 years	222 (64.2)	114 (86.4)	
>60 years	124 (35.8)	18 (13.6)	
BMI (kg/m^2^)			0.666
< 18.5	57 (16.5)	24 (18.2)	
≥ 18.5	288 (83.5)	108 (81.8)	
Tobacco smoking			0.860
Never	244 (70.5)	92 (69.7)	
Ever	102 (29.5)	40 (30.3)	
Alcohol drinking			0.395
Never	257 (74.3)	103 (78.0)	
Ever	89 (25.7)	29 (22.0)	
Education			< 0.001
Illiterate or semi-illiterate	35 (11.2)	5 (3.8)	
Primary school	95 (30.5)	11 (8.4)	
Middle school	94 (30.1)	23 (17.4)	
High school	54 (17.3)	37 (28.0)	
University or college education	34 (10.9)	56 (42.4)	
Marriage status			< 0.001
Unmarried	60 (18.9)	44 (33.3)	
Married	235 (74.1)	87 (65.9)	
Divorced or widowed	22 (7.0)	1 (0.8)	
Economic level			0.220
Top	46 (13.8)	15 (11.4)	
Middle	101 (30.3)	51 (38.6)	
Bottom	186 (55.9)	66 (50.0)	
Occupational status			< 0.001
Farmer	230 (66.5)	25 (18.9)	
Laborer/sales/housekeeping	59 (17.0)	60 (45.5)	
Professional/supervisory/technical	57 (16.5)	47 (35.6)	
Diabetes mellitus			0.807
No	312 (90.2)	120 (90.9)	
Yes	34 (9.8)	12 (9.1)	
ATT history			0.013
No	327 (94.5)	116 (87.9)	
Yes	19 (5.5)	16 (12.1)	
Treatment regimen			0.002
2HRZE/4HR	334 (96.5)	118 (89.4)	
Others	12 (3.5)	14 (10.6)	
Treatment compliance			0.335
Good	333 (96.2)	130 (98.5)	
Poor	13 (3.8)	2 (1.5)	

^*^: Comparison was used by chi-square test or calibration chi-square test

Abbreviations: BMI, body mass index; ATT, antituberculosis treatment; 2HRZE/4HR: H, isoniazid; R, rifampin; Z, pyrazinamide; E, ethambutol

**Table 2:** Baseline laboratory tests and clinical indicators

Characteristics	Training cohort	Validation group	*P*
Sputum smear, n (%)			0.350
0 Scanty or 1+ 2+ 3+	240 (69.4) 57 (16.5) 17 (4.9) 32 (9.2)	86 (65.1) 20 (15.2) 12 (9.1) 14 (10.6)	
Pulmonary cavity, n (%)			0.001
Absence Presence	208 (60.1) 138 (39.9)	58 (43.9) 74 (56.1)	
CXR score, Median (IQR)	33.33 (40)	25 (40)	< 0.001
Symptom score, Mean ± SD	3.39 ± 1.62	2.14 ± 1.29	< 0.001
Delayed treatment (days), Median (IQR)	47 (71)	30 (83)	< 0.001
Red blood cell (×10^12^/L), Mean ± SD	4.68 ± 0.58	4.61 ± 0.59	0.288
White blood cell (×10^9^), Median (IQR)	6.3 (5.3)	5.94 (2.54)	0.012
Platelet (×10^9^/L), Mean ± SD	238.77 ± 99.46	252.27 ± 86.60	0.218
Hemoglobin (g/L), Mean ± SD	135.90 ± 18.57	130.82 ± 16.48	0.014
Packed cell volume (L/L), Mean ± SD	0.411 ± 0.054	0.371 ± 0.057	< 0.001
Red blood cell distribution width (%), Mean ± SD	13.94 ± 1.56	13.02 ± 1.47	< 0.001
Absolute value of lymphocytes (×10^9^/L), Mean ± SD	1.44 ± 0.50	1.61 ± 0.52	0.003
Lymphocytes percentage (%), Mean ± SD	22.55 ± 9.14	28.21 ± 9.71	< 0.001
Monocyte absolute value (×10^9^/L), Mean ± SD	0.55 ± 0.27	0.45 ± 0.17	< 0.001
Monocyte percentage (%), Mean ± SD	7.74 ± 2.61	7.51 ± 2.15	0.399
Absolute value of neutrophil (×10^9^/L), Mean ± SD	4.91 ± 2.75	3.85 ± 1.82	< 0.001
Neutrophilic percentage (%), Mean ± SD	65.98 ± 12.31	61.00 ± 10.64	< 0.001
Absolute value of eosinophils (×10^9^/L), Median (IQR)	0.1 (0.13)	0.14 (0.15)	0.009
Eosinophil percentage (%), Median (IQR)	1.6 (2)	2.50 (2.35)	< 0.001
Absolute value of basophils (×10^9^/L), Median (IQR)	0 (0.01)	0.02 (0.01)	< 0.001
Basophil percentage (%), Median (IQR)	0.4 (0.3)	0.4 (0.2)	0.212
Total bilirubin (µmol/L), Median (IQR)	10.54 ± 8.43	11.05 ± 8.62	0.684
Alanine aminotransferase (U/L), Median (IQR)	24.80 (16.1)	15.00 (11.00)	< 0.001
Aspartate aminotransferase (U/L), Median (IQR)	25.88 (13.3)	17.05 (8.25)	< 0.001
Urea (mmol/L), Mean ± SD	4.67 ± 1.68	4.08 ± 1.27	< 0.001
Creatinine (µmol/L), Mean ± SD	67.78 ± 17.87	63.83 ± 12.18	0.037
Uric acid (µmol/L), Mean ± SD	415.38 ± 192.10	394.83 ± 149.85	0.292
Cystatin C (mg/L), Mean ± SD	1.06 ± 0.31	1.25 ± 0.48	< 0.001
β2-microglobulin (mg/L), Mean ± SD	2.40 ± 0.88	1.40 ± 0.41	< 0.001

Abbreviations: CXR, chest x-ray; IQR, interquartile range; SD, standard deviation

We enrolled 132 TB patients in the second stage as the validation population. Detailed inclusion and exclusion criteria are shown in the diagram in Fig. 1B. Among them, 78 (59.1%) were males, 18 (13.6%) were over 60 years, 24 (18.2%) were underweight, 40 (30.3%) were ever-smokers, and 29 (22.0%) had an alcohol drinking history. There were 16 (12.1%) subjects who had an ATT history, and 12 (9.1%) were diagnosed with diabetes mellitus. There were 25 (18.9%) farmers, 60 (45.5%) patients working as laborers/sales/housekeeping, and 66 (50.0%) were low-income. Most of them received 6-month treatment with four first-line drugs, and only 2 patients had poor drug compliance (Table 1). Laboratory results of the study subjects are described in Table 2. The average follow-up time was 185 days, and we finally observed 15 patients with unfavorable outcomes, incorporating 2 treatment failures, 7 adverse drug reactions, 3 transferring to multidrug-resistant therapy, and 3 relapses.

We also compared demographics and laboratory values between two cohort populations (Tables 1 and 2). Results showed significant differences in sex, age, education levels, marriage status, occupational status, ATT history, and treatment regimens between the training cohort and validation population. Besides, there were meaningful differences in the pulmonary cavity, CXR score, symptom score, delayed treatment, and 18 clinical indicators. Overall, there existed heterogeneities in the two study populations.

### Clinical indicator-based prognostic model

We performed a LASSO Cox regression analysis on 24 clinical indicators in the treatment outcomes of patients with TB. Eventually, 10 indicators (PLT, PCV, LYMPH, MONO%, NEUT, NEUT%, TBTL, ALT, UA, and Cys-C) remained in the final model. The coefficients of these indicators were utilized to calculate the risk score as follows: risk score = PLT×0.8236- PCV×0.6823- LYMPH×0.4442- MONO%×0.2720 + NEUT×0.0222 + NEUT%×0.0027- TBTL×0.5511- ALT×0.6839 + UA×0.6198 + Cys-C×0.6040. The univariate Cox regression analysis showed that the HR of the risk score was 4.980 (95% CI: 3.030–8.185, *P* < 0.001).

**Fig. 2:**
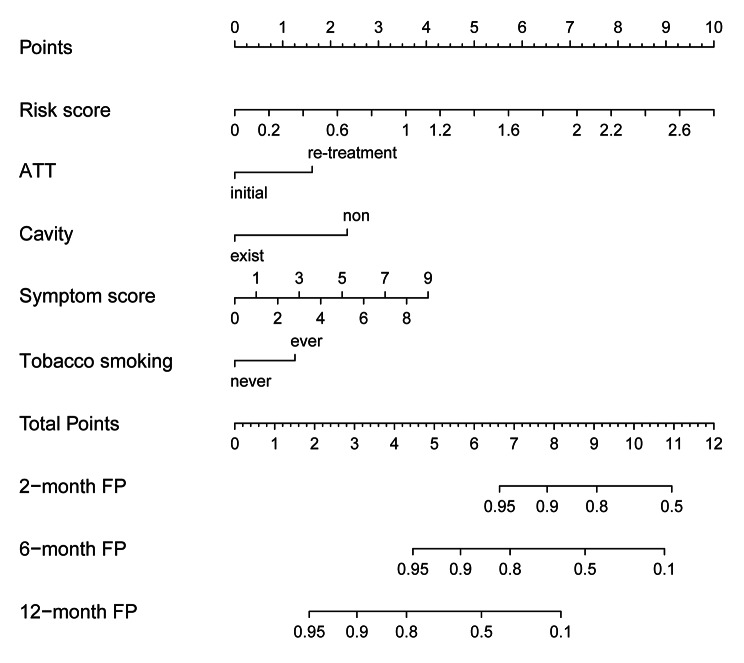
** A risk score nomogram predicts the favorable prognosis of patients with tuberculosis** First, we locate the risk score on the risk score axis and draw a vertical line up to the points axis to identify how many points the risk score contributes to a favorable prognosis (FP). Then, we use the same method for ATT history, pulmonary cavity, symptom score, and smoking. The total points are the sum of each factor. Finally, we locate the patient’s total points on the total points axis and draw a vertical line down to the probability of a 2-month, 6-month, and 12-month favorable prognosis ATT: antituberculosis treatment; FP: favorable prognosis

We further analyzed the effects of patient characteristics on the treatment outcomes (Table 3). Results showed that patients over 60 years, illiterate or semi-illiterate, married, with previous treatment history, treated with other regimens except for 2HRZE/4HR, with treatment interruptions, and with higher symptom scores were associated with an adverse outcome. Then, we performed a multivariate Cox regression analysis on the risk score by considering sex, age, BMI, tobacco smoking, alcohol drinking, education, marriage status, economic level, occupational status, previous medical history, ATT, treatment regimen, treatment compliance, sputum smear test at the time of diagnosis, CXR score, pulmonary cavity, symptom score, and delayed treatment (Table 4). Finally, the risk score (HR: 10.018, 95% CI: 4.904–20.468, *P* < 0.001), symptom score (HR: 1.356, 95% CI: 1.079–1.704, *P* = 0.009), pulmonary cavity (HR: 0.242, 95% CI: 0.087–0.674, *P* = 0.007), ATT (HR: 2.810, 95% CI: 1.137–6.948, *P* = 0.025), and tobacco smoking (HR: 2.499, 95% CI: 1.097–5.691, *P* = 0.029) constructed the optimal model. We graphed a nomogram based on this model to predict the 2-month, 6-month, and one-year favorable prognosis probability (Fig. 2).

**Table 3:** Univariate Cox regression analysis of demographic factors and laboratory results

Variables	HR (95% CI)	*P*
Sex		
Males	1	
Females	0.983 (0.419, 2.307)	0.969
Age		
≤60 years	1	
> 60 years	2.425 (1.185, 4.960)	0.015
BMI		
<18.5 kg/m^2^	1	
≥18.5 kg/m^2^	1.063 (0.453, 2.495)	0.887
Tobacco smoking		
Never	1	
Ever	1.133 (0.560, 2.291)	0.729
Alcohol drinking		
Never	1	
Ever	0.930 (0.444, 1.946)	0.846
Education		
Illiterate or semi-illiterate	1	
Primary school	0.489 (0.179, 1.337)	0.164
Middle school	0.424 (0.148, 1.212)	0.109
High school	0.368 (0.109, 1.247)	0.108
University or college education	0.460 (0.091, 2.316)	0.346
Marriage status		
Unmarried	1	
Married	2.692 (0.634, 11.420)	0.179
Divorced or widowed	2.274 (0.317, 16.260)	0.413
Economic level		
Top	1	
Middle	1.868 (0.396, 8.804)	0.429
Bottom	1.518 (0.343, 6.717)	0.582
Occupational status		
Farmer	1	
Laborer/sales/housekeeping	0.544 (0.187,1.581)	0.263
Professional/supervisory/technical	1.754 (0.685, 4.490)	0.242
Diabetes mellitus		
No	1	
Yes	1.529 (0.648, 3.617)	0.332
ATT history		
No	1	
Yes	2.212 (0.911, 5.367)	0.079
Treatment regimen		
2HRZE/4HR	1	
Others	2.314 (0.846, 6.333)	0.102
Treatment compliance		
Good	1	
Poor	3.194 (1.087, 9.381)	0.035
Sputum smear at diagnosis		
0	1	
Scanty or 1+	0.618 (0.225, 1.696)	0.351
2+	1.127 (0.364, 3.487)	0.836
3+	0.725 (0.241, 2.183)	0.568
Pulmonary cavity		
Absence	1	
Presence	0.832 (0.393, 1.762)	0.631
CXR score	1.001 (0.989, 1.014)	0.876
Symptom score	1.231 (0.992, 1.529)	0.060
Delayed treatment	1.000 (0.997, 1.003)	0.800
Risk score	4.980 (3.030, 8.185)	< 0.001

Abbreviations: HR, hazard ratio; CI, confidence interval; ATT, antituberculosis treatment; CXR, chest x-ray; 2HRZE/4HR: H, isoniazid; R, rifampin; Z, pyrazinamide; E, ethambutol

**Table 4:** Multivariate Cox regression analysis on the prognosis of patients with tuberculosis

Variables	HR (95% CI)	*P*
Risk score	10.018 (4.904, 20.468)	< 0.001
Symptom score	1.356 (1.079, 1.704)	0.009
Pulmonary cavity		
Absence	1	
Presence	0.242 (0.087, 0.674)	0.007
ATT history		
No	1	
Yes	2.810 (1.137, 6.948)	0.025
Tobacco smoking		
Never	1	
Ever	2.499 (1.097, 5.691)	0.029

Abbreviations: HR, hazard ratio; CI, confidence interval; ATT, antituberculosis treatment

### Models evaluation

**Fig. 3:**
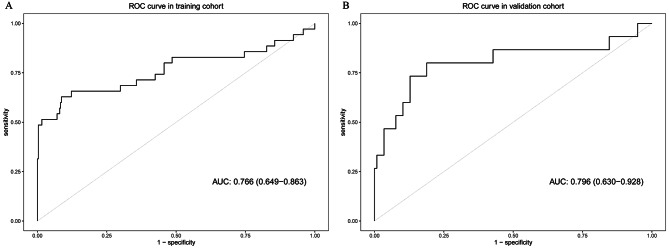
**ROC curves for the multivariate prognosis models** ROC curves of clinical indicator-based prognostic models in training cohort (A) and validation cohort (B) ROC: receiver operating characteristic

The C-index was 0.709 (95% CI: 0.548–0.870) for the univariate risk score model and 0.783 (95% CI: 0.675–0.891) for the multivariate prognostic model. The Akaike information criterion (AIC) was 271.230 for the univariate model and 265.653 for the multivariate model. Hosmer-Lemeshow tests indicated a good fit for these two models (*P* > 0.05). We plotted ROC curves to evaluate the predictive ability and calculated the corresponding AUC values (Fig. 3). The AUC was 0.766 (95% CI: 0.649–0.863) for the multivariate model in the training cohort and 0.796 (95% CI: (0.630–0.928) for the multivariate model in the external validation population. Internal validation showed an AUC of 0.763 (95% CI: 0.758–0.768) for the multivariate model.

### Sensitivity analysis and subgroup analysis

Considering that the pulmonary cavity may affect the results, we performed a sensitivity analysis to test the consistency of this association by adding delayed time into the final prognosis model. Results showed that the pulmonary cavity remained a protective factor for the prognosis of TB (Supplementary Table 2).

**Fig. 4:**
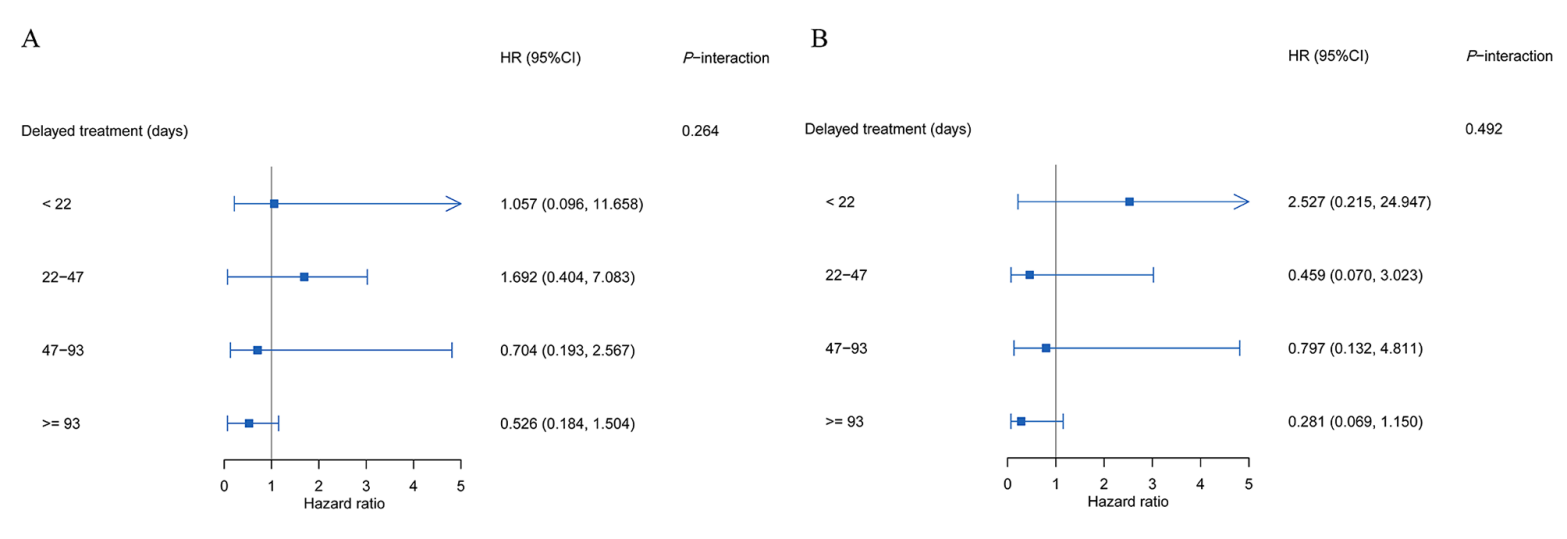
**Forest plots of the effect of the pulmonary cavity on the prognosis of patients with tuberculosis** Forest plots depicting the association between the pulmonary cavity and TB prognosis in the subgroup of treatment delay using univariate regression model (A) and multivariate regression model (B) HR: hazard ratio; CI: confidence interval

Furthermore, we divided the delayed time into four groups by quartiles (25, 50, 75) and investigated its modification. In the univariate and multivariate models, no significant association was observed between the cavity and TB prognosis in each subgroup (Fig. 4).

## Discussion

In the present study, we developed and validated a prognosis prediction model for patients with TB. Results showed that the clinical indicators-based risk score was significantly associated with the treatment outcomes. Findings from this study provide more references for the prognosis prediction of TB.

Previous studies have utilized different scoring systems to predict the prognosis. For example, Holden et al. constructed the CCIs-based model by considering cardiovascular disease, liver and kidney disease, mental disease, metabolic disease, and tumor [16]. Colangeli et al. calculated the Karnofsky scores to classify functional impairment to assess patients’ prognosis [15]. Nonetheless, as additional predictive tools, the aforementioned score systems relied on the patient’s recall and doctor’s judgment, prone to information bias. Blood tests and biochemistry examinations are routinely applied in clinical settings, providing accessible indicators. Therefore, we constructed a clinical indicator-based risk score to predict the treatment outcomes of TB by using PLT, PCV, LYMPH, MONO%, NEUT, NEUT%, TBTL, ALT, UA, and Cys-C.

A newly published study suggested that neutrophil, neutrophilic percentage, and neutrophil to lymphocyte ratio (NLR) were significantly related to different lung involvements among COVID-19 patients. The combination of NLR, lactate dehydrogenase, glucose, and ALT worked best to ascertain the clinical stage of COVID-19 [23]. Luo et al. established clinical indicators in routine blood tests to distinguish between active TB and latent tuberculosis infection (LTBI) [24]. Stefanescu et al. supposed that inflammatory biomarkers, including CRP, WBC, neutrophils, interferon-gamma inducible protein 10, CRP to albumin ratio (CAR), neutrophil to albumin ratio (NAR) and serum LL37, had a good prediction ability for 2-months treatment outcomes of pulmonary TB patients [25]. Previous studies also suggested that the power of a single index to predict prognosis was limited, and the combination of clinical indicators can effectively improve the prediction effectiveness.

The onset of TB always presented several clinical symptoms, including cough, expectoration, fever, weight loss, dyspnea, night sweats, hemoptysis, fatigue, and chest pain, which were closely linked to the severity of the disease. Our results uncovered the number of symptoms was positively related to the poor prognosis of TB patients, indicating that clinical symptoms and signs are associated with bacterial burden, infection site, and host immune response and thus affect treatment outcomes [26].

We found that patients with pulmonary cavities were inclined to have favorable outcomes, which seemed to be wired. This may be mediated by the fact that typical radiography findings are beneficial for accurate TB diagnosis. The discovery was in line with other studies. A cohort study has proposed atypical imaging features and sputum smear-negative at diagnosis were strongly related to delayed isolation and treatment that may cause an unfavorable prognosis [27]. Another study for hospitalized TB patients showed that noncavitary imaging manifestation might lead to misdiagnosis or delayed diagnosis, resulting in increased mortality [28]. However, it was important to note that similar radiological findings could observe in other diseases such as lung abscess, lung cancer, and pneumonia. Other characteristics like tree-in-bud appearance may be further considered. Besides, it should be noted that when patients received ATT during a relatively short period, the existence of a cavity may also be considered a risk factor for the favorable prognosis, which was shown in the subgroup analyses; this was supported by the results of Koo et al. [29]. It needs to be verified by further research through the expanded sample size.

Some studies have demonstrated that recurrent TB was a risk factor for the development and prognosis of TB [9, 14]. A nested case-control study in Vietnam elucidated that ATT history played a crucial role in the recurrence of TB, mainly due to increased drug resistance [30]. Poor treatment adherence and inadequate antibiotic therapy may also cause incomplete eradication of the causative *Mycobacterium tuberculosis (M.tb)* [31, 32].

Previous studies have reported that smokers were more prone to adverse treatment outcomes than non-smokers, which was consistent with our findings [33–35]. Harmful materials in cigarettes, like nicotine, could directly impair the human immune system and weaken the ability to kill *M.tb* in vivo [36]. Moreover, exposure to tobacco smoke would damage the respiratory tract, which constitutes early host defense against bacteria, thereby negatively affecting immunity [37].

However, there are several limitations to this study. First, this study was performed with a limited sample size in Jiangsu, China. Thus, caution should be exercised in extrapolating research results to other regions. Second, in this study, we excluded HIV-positive TB patients during the recruitment. Considering the critical role of HIV infections in pulmonary TB treatment effectiveness, we should bring this risk factor into further study. Third, we did not collect information about past medication history and daily ability to function, which could influence the prognosis of TB. It will be improved in future studies. Last, we only collected the baseline clinical data before treatment to calculate the risk score. It may neglect the importance of continuous follow-up and management. Future studies should consider the impact of dynamic changes in leading clinical indicators during follow-up on the prognosis, which will give more scientific justification.

In summary, we systematically generated a risk score-based model by integrating routine clinical information and demographic characteristics to predict ATT outcomes with feasibility and rationality in the clinic. Though there were population heterogeneities between the two cohorts, external validation remained a good performance of the model showing a relatively reasonable extrapolation.

## Conclusion

In addition to the traditional predictive factors, the clinical indicator-based risk score determined in this study has an excellent predictive effect on the prognosis of TB.

## Electronic supplementary material

Below is the link to the electronic supplementary material.

Supplementary Material 1

## Data Availability

All data generated or analyzed during this study are included in this published article.

## Abbreviations

AIC: Akaike information criterion
ALT: Alanine aminotransferase
AST: Aspartate aminotransferase
ATT: Antituberculosis treatment
AUC: Area under the curve
BASO: Basophils
BMI: Body mass index
CCI: Charlson comorbidity index
CI: Confidence interval
Cr: Creatinine
CXR: Chest X-ray
Cys-C: Cystatin C
DM: Diabetes mellitus
EOS: Eosinophils
HB: Hemoglobin
HIV: human immunodeficiency virus
HR: Hazard ratio
IQR: Interquartile range
LASSO: Least absolute shrinkage and selection operator
LTBI: Latent tuberculosis infection
LYMPH: Lymphocytes
MONO: Monocyte
*M.tb*: *Mycobacterium tuberculosis*
NEUT: Neutrophil
NLR: Neutrophil to lymphocyte ratio
PCV: Packed cell volume
PLT: Platelet
RBC: Red blood cell
RDW: Red blood cell distribution width
ROC: Receiver operating characteristic
SD: Standard deviation
TB: Tuberculosis
TBTL: Total bilirubin
UA: Uric acid
UREA: Urea
WBC: White blood cell
β2-m: β2-microglobulin
