# Supplementary material for: A clinical indicator-based prognostic model predicting treatment outcomes of pulmonary tuberculosis: a prospective cohort study
Source: BMC Infect Dis. 2023 Feb 20;23:101. doi: 10.1186/s12879-023-08053-x (PMC9940065; doi:10.1186/s12879-023-08053-x)
Supplement: Supplementary file 1 — Supplementary Material 1 [file 12879_2023_8053_MOESM1_ESM.docx]

**Supplementary material**

**1 Missing data imputation**

**1.1 Descriptive statistics**

Detailed missing data of clinical indicators were reported in Supplementary Table 1. The percentage of missing data ranged from a low of 4.0% to a high of 28.9%. Overall, 29.8% of subjects had missing data of at least 1 variable.

Supplementary Table 1. Detailed missing data of clinical indicators.

| Variable | No. of subjects with observed data | No. of subjects with missing data | Percentage of subjects with missing data |
| --- | --- | --- | --- |
| Red blood cell (×10^12^/L) | 246 | 100 | 28.9% |
| White blood cell (×10^9^) | 246 | 100 | 28.9% |
| Platelet (×10^9^/L) | 246 | 100 | 28.9% |
| Hemoglobin (g/L) | 247 | 99 | 28.6% |
| Packed cell volume (L/L) | 246 | 100 | 28.9% |
| Red blood cell distribution width (%) | 244 | 102 | 29.5% |
| Absolute value of lymphocytes (×10^9^/L) | 246 | 100 | 28.9% |
| Lymphocytes percentage (%) | 246 | 100 | 28.9% |
| Monocyte absolute value (×10^9^/L) | 243 | 103 | 29.8% |
| Monocyte percentage (%) | 243 | 103 | 29.8% |
| Absolute value of neutrophil (×10^9^/L) | 246 | 100 | 28.9% |
| Neutrophilic percentage (%) | 246 | 100 | 28.9% |
| Absolute value of eosinophils (×10^9^/L) | 243 | 103 | 29.8% |
| Eosinophil percentage (%) | 243 | 103 | 29.8% |
| Absolute value of basophils (×10^9^/L) | 243 | 103 | 29.8% |
| Basophil percentage (%) | 243 | 103 | 29.8% |
| Total bilirubin (μmol/L) | 332 | 14 | 4.0% |
| Alanine aminotransferase (U/L) | 332 | 14 | 4.0% |
| Aspartate aminotransferase (U/L) | 332 | 14 | 4.0% |
| Urea (mmol/L) | 282 | 64 | 18.5% |
| Creatinine (μmol/L) | 282 | 64 | 18.5% |
| Uric acid (μmol/L) | 281 | 65 | 18.8% |
| Cystatin C (mg/L) | 261 | 85 | 24.6% |
| β2-microglobulin (mg/L) | 261 | 86 | 24.6% |

**1.2 Comparison of subjects with and without missing data**

We conducted univariate comparisons of those with and without missing data which will provide evidence as to the plausibility of the missing at random (MAR) assumption. We found a significant difference in age. The average age of those with complete data was 52.8 years, and 50.8 years for those with missing data. Patients with missing data tended to be younger than those with complete data.

**1.3 Descriptive statistics in the imputed data sets**

Predictive mean matching (PMM) was chosen as the imputation method for 24 clinical indicators. Red blood cell, for example, is depicted in Supplementary Figure 1. The density function in the complete cases is shown as a blue line, and the density function of the imputed variable in each of the imputed data sets is shown with a red line. The distribution of the imputed values tended to be very similar to that of the observed values of the variable.

**
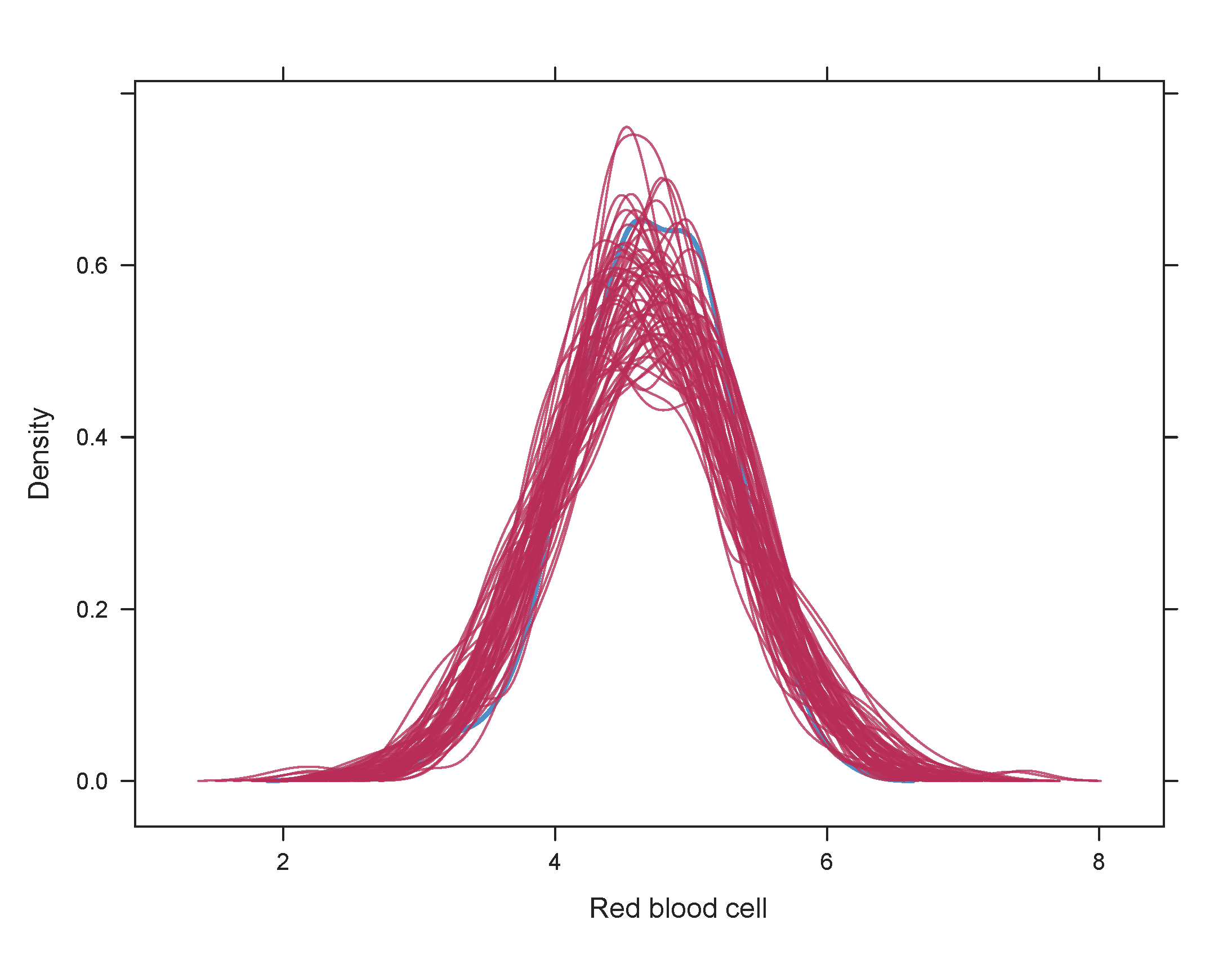
**

**Supplementary Figure 1. Distribution of red blood cell values in complete cases and those with imputed data when using predictive mean matching (PMM).**

The blue line denotes the distribution of the given continuous variable in those subjects for whom that variable was not missing. The red lines represent the distribution of the imputed value for that variable in those subjects for whom the variable was missing.

**2 Sensitivity analysis**

Supplementary Table 2. Multivariate Cox regression analysis by considering delayed treatment.

| Variables | HR (95% CI) | *P* |
| --- | --- | --- |
| Risk score | 9.995 (4.890, 20.431) | <0.001 |
| Symptom score | 1.356 (1.080, 1.702) | 0.009 |
| Delayed treatment | 1.000 (1.000, 1.003) | 0.846 |
| Pulmonary cavity |  |  |
| Absence | 1 |  |
| Presence | 0.242 (0.087, 0.674) | 0.007 |
| ATT history |  |  |
| No | 1 |  |
| Yes | 2.793 (1.127, 6.918) | 0.026 |
| Tobacco smoking |  |  |
| Never | 1 |  |
| Ever | 2.513 (1.101, 5.737) | 0.029 |
